# Aboriginal and Torres Strait Islander children and cancer: a narrative review of incidence, mortality, barriers to diagnosis and treatment, psychosocial needs and interventions

**DOI:** 10.1016/j.lanwpc.2025.101530

**Published:** 2025-03-30

**Authors:** Samantha Bay, Emma V. Taylor, Melanie Robinson, Leanne Pilkington, Sandra C. Thompson

**Affiliations:** a Western Australian Centre for Rural Health, University of Western Australia, 167 Fitzgerald St, Geraldton, Western Australia, 6531, Australia; b Child and Adolescent Health Service, Department of Health Western Australia, 15 Hospital Ave, Nedlands, Western Australia, 6009, Australia

**Keywords:** Cancer, Oncology, Children, Paediatric, Indigenous, Aboriginal, Torres Strait Islander

## Abstract

Compared to adult cancer in Aboriginal and Torres Strait Islander populations, minimal research has focussed on cancer in Indigenous Australian children. This narrative review examined published information about incidence, mortality, barriers to diagnosis and treatment, and psychosocial needs and interventions for Indigenous Australian children with cancer. Most papers were epidemiological, investigating incidence and mortality. A reported lower overall cancer incidence in Indigenous Australian compared to non-Indigenous children may reflect detection bias. Some studies suggested differences in the incidence of types of cancers. There were conflicting findings about differences in mortality rates. Reported barriers to diagnosis and treatment include individual, systemic and cultural factors. There was a lack of published studies about psychosocial needs and interventions for Indigenous Australian children with cancer. Further research is needed to better understand complexities in the presentation of cancer in Indigenous Australian children and identify their psychosocial needs to ensure interventions are relevant and holistic.

**Research in context**

**Evidence before this study**

• Compared to adult cancer in Aboriginal and Torres Strait Islander populations, minimal research has focussed on cancer in Indigenous Australian children.
• A review on cancer in First Nations' children internationally was published a decade ago.

**Added value of this study**

• This review identified that most papers were epidemiological. Research about psychosocial needs and interventions for Indigenous Australian children with cancer is lacking.
• This review discussed the gaps in the literature and made recommendations for future research to address.

**Implications of all the available evidence**

• Future research should seek to understand the intersecting biological, environmental, behavioural, and psychological factors that influence outcomes of cancer in Indigenous Australian children.
• Given the challenges identified in this paper of conducting research with Indigenous Australian children, a collaborative approach across the country may be a helpful strategy to overcome the limitations of small samples.
• Achieving better cancer outcomes for Indigenous Australian children requires co-designed and collaborative research projects.

## Introduction

Aboriginal and Torres Strait Islander peoples (hereafter respectfully referred to as Indigenous) have been documented to have higher rates of cancer and have a lower median age of incidence compared to non-Indigenous Australians.^1^ However, there has been less attention on childhood cancers in Indigenous Australians with most articles and reports focused on adult incidence, issues, and treatment. It has been shown that childhood cancers differ from adult cancers in aetiology, biology, and treatment.^2^^,^^3^ For example, in contrast to adult treatments, most treatments for childhood cancers aim to cure rather than to palliate.^3^ Unlike adult cancers, most childhood cancers are not associated with modifiable risk factors; therefore, improving outcomes requires accurate earlier diagnosis and effective treatments.^4^^,^^5^ As the incidence rate of cancer in childhood in Australia increased by 1.2% per year between 2005 and 2015, and rates are expected to continue rising,^6^ it is important to identify current knowledge and gaps to optimise and plan for cancer care for children and their families.

A review on cancer in First Nations’ children internationally was published a decade ago,^7^ and to our knowledge there are no published reviews specifically on cancer in Indigenous Australian children. This paper summarises and discusses the current literature on cancer in Indigenous Australian children and identifies gaps that could inform future research. Specifically, this paper explores the following questions: 1) What differences, if any, are there in cancer incidence between Indigenous Australian children and non-Indigenous children?; 2) What are the differences, if any, in mortality rates between Indigenous Australian children and non-Indigenous children with cancer?; 3) What are the barriers to receiving diagnosis and treatment for Indigenous Australian children with cancer?; 4) What psychosocial needs have been identified in the literature, and have interventions been able to meet these needs for Indigenous Australian children with cancer?

## Methods

This narrative review was conducted in accordance with best practice recommendations for narrative reviews in clinical research.^8^ A systematic search was conducted, followed by title and abstract, and full text screenings. Information from studies was manually extracted by authors using Covidence data extraction tool “Extraction 2”.^9^ Authors examined the data and summarised findings in this review.

### Search strategy and selection criteria

A literature search was conducted between the 9th and 14th of May 2024, using database-specific search strings across the following databases: PubMed, CINAHL, PsycINFO, Australian Indigenous Health InfoNet, and Google Scholar. Key search terms “Aboriginal” and/or “Indigenous”, and “paediatric” and/or “child∗”, and “cancer” and/or “oncology” were searched using a combination of subject headings and free text keywords. A full list of the search terms used for each database are displayed in Appendix A. Children were defined in this paper as those aged under 18 years. Studies with samples that included a combination of children and young adults were included, while studies with whole populations (i.e., across the lifespan) or had samples predominantly consisting of adults were excluded.

Authors also manually checked the reference lists of included articles for additional relevant studies. Only peer-reviewed papers that explored questions about childhood cancer in Australia that were published in English were reviewed. Given the limited number of studies exploring childhood cancer in Indigenous Australian children, there was no exclusion based on date of publication for peer-reviewed articles.

Two reviewers (SB and EVT) independently screened titles and abstracts of publications, and then independently reviewed those requiring full text assessment. Articles were excluded following full text examination if the study was about other childhood diagnoses that were not cancer, study sample consisted of Indigenous Australian adults only, study sample did not include Indigenous Australian children, study was a review article, and study sample was across the lifespan but consisted predominantly of adults. Discrepancies were discussed and resolved by consensus. All authors were consulted to identify any further studies for inclusion and agreed on a consensus approach to analysis. Information on cancer incidence, mortality, barriers to diagnosis and treatment, psychosocial needs and interventions were extracted and qualitatively synthesised. Articles with a stated aim of investigating cancer in Indigenous Australian children or which made substantial findings related to cancer in Indigenous Australian children were noted, as some studies were primarily about other topics but included data about cancer in Indigenous Australian children.

Where literature was lacking to explain concepts relating to childhood cancer in Indigenous Australian children, Indigenous Australian adult research or papers from overseas were drawn upon to allow for discussion and inference about directions for future research.

## Results

**Fig. 1:**
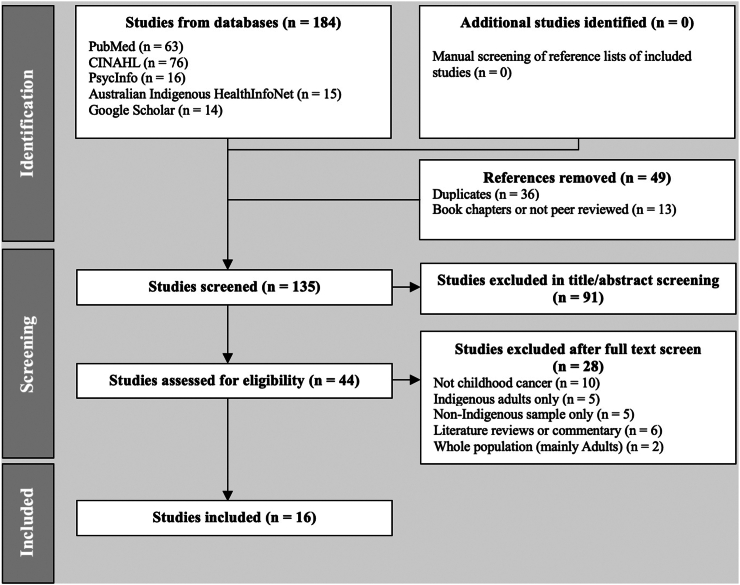
**Summary of the process of identification, screening, and inclusion of studies**.

**Fig. 2:**
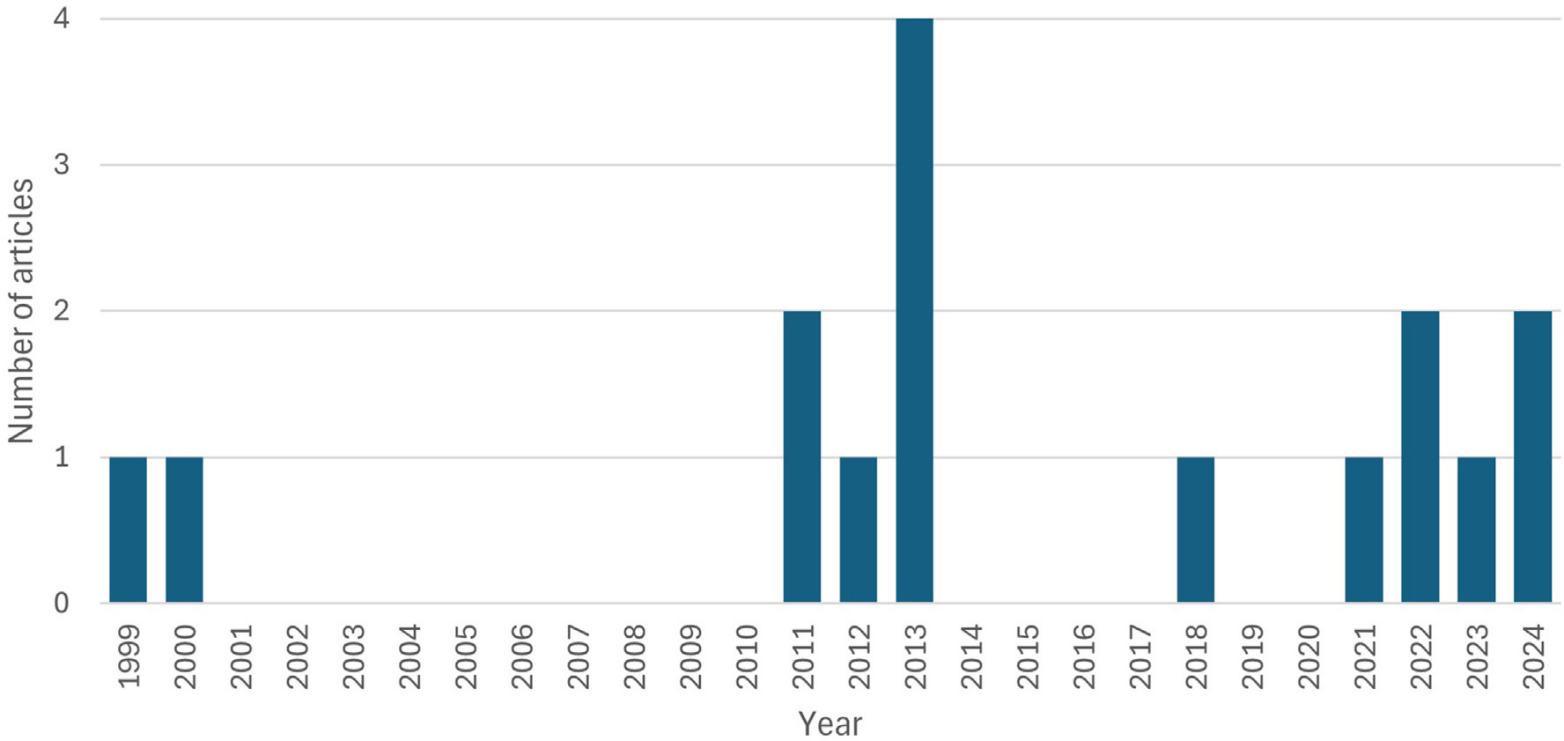
**Number of articles on cancer in Indigenous Australian children by year of publication∗**. **∗Note: Search was conducted in May 2024, so 2024 does not represent the whole year**.

We screened 135 papers and identified 16 with findings on cancer in Indigenous Australian children across the 25-year period 1999 to 2024 (Fig. 1, Tables 1 and 2 summarise papers identified). We noted a substantial increase in the number of papers over time, with a cluster of articles published between 2011 and 2013 (n = 7, 44%) and six (38%) published in the last four years (Fig. 2).

**Table 1:** Epidemiological studies.

Authors (Year of publication) location of study	Indigenous Australian childhood cancer focus^a^	Source of data	Age group (years)	Date of study data	Number of Indigenous Australian children	Study aim	Relevant topics addressed in this review
Abdalla et al. (2023)^10^ WA	No	WA datasets	0–18	1982–2019	138 cancer (4.7%), 1523 comparisons (6.1%)	Examine hospitalization trends for physical diseases and estimate the associated inpatient care costs in all 5-year CCS diagnosed in WA from 1982 to 2014.	Other: Rate of hospitalisation
Alessandri et al. (1999)^11^ WA	No	WA datasets	1–6	1981–1996	88 deaths (17.5%); 3 cancer- related (0.6%)	Investigate childhood mortality for all children aged 1–6 years inclusive born from 1980 to 1989 and dying from 1981 to 1996 in WA.	Mortality
Azzopardi et al. (2018)^12^ Australia wide	No	National datasets	10–24	2004–2013	Not stated	Report a national health profile for Indigenous adolescents in Australia.	Incidence
Bartle & Rice (2000)^13^ NT, SA, Victoria	Yes	Hospital records	0–16	1968–1998	8 (18%)	Audit AML cases diagnosed and treated at the Adelaide Women's and Children's Hospital over the years 1968–1998, and report on the overrepresentation of Indigenous Australians in the data.	Incidence
Haggar et al. (2013)^14^ WA	No	WA datasets	15–39	1982–2004	821 (8%)	Model survival and excess mortality in all adolescents and young adults aged 15–39 years in WA with a diagnosis of cancer in the period 1982–2004.	Mortality
Jessop et al. (2021)^15^ NT, SA, WA	Yes	Hospital records	0–18	2009–2018	29 (6.4%)	Compare presentation patterns, follow-up and clinical outcomes in Indigenous and non-Indigenous children with acute leukaemia in Australia, and to assess the impact of remoteness and area-based socioeconomic disadvantage on outcome.	Incidence Mortality Barriers Other: Clinical trials
Mashtoub et al. (2024)^16^ NT, SA	Yes	State Cancer Registries (NT & SA)	0–19	1990–2017	39 (28%)	Determine the age and incidence of childhood cancers (leukemias and embryonal tumours) and characterize the trends in overall survival in the period 1990–2017 in SA and the NT. Gain insight into the trends and overall survival amongst Indigenous Australian children with cancer in the NT.	Incidence Mortality
Rotte et al. (2013)^17^ NT, SA	Yes	Hospital records	0–18	1997–2011	41 (36%)	Investigate cancer in Indigenous Australian children in SA and NT. Determine whether there are differences in cancer presentation, distribution and outcome between Indigenous Australian and non–Indigenous children.	Incidence Mortality Barriers Other: Clinical trials Other: Length of hospital stay
Valery et al. (2013)^18^ Australia wide	Yes	Australian Childhood Cancer Registry	0–14	1997–2008	224 (3%)	Examine the national patterns of incidence and mortality for cancers diagnosed amongst Indigenous Australian children and compare with non-Indigenous children.	Incidence Mortality
Valery et al. (2013)^19^ Australia wide	Yes	Australian Childhood Cancer Registry	0–14	1997–2007	196 (3%)	Quantify the extent of, and understand reasons for, any survival differential for Indigenous Australian childhood cancer patients	Mortality
Youlden et al. (2011)^20^ Australia wide	No	Australian Childhood Cancer Registry	0–14	1996–2006	Not stated	Examine and quantify the effects of remoteness of residence and an area-based measure of socioeconomic disadvantage on childhood cancer survival in Australia.	Mortality
Youlden et al. (2012)^21^ Australia wide	No	Australian Childhood Cancer Registry	0–14	1996–2006	785^b^ (11.6%)	Investigate the association between different types of childhood cancer and an area-based measure of socioeconomic status. Examine whether there was a link between remoteness of residence and the incidence of childhood cancer.	Incidence
Youlden et al. (2021)^22^ Australia wide	Yes	Australian Childhood Cancer Registry	0–14	1997–2017	506 (3.8%)	Determine the current rates of cancer incidence and survival among Indigenous Australian children and describe trends over the last two decades.	Incidence Mortality

AML, Acute Myeloid Leukaemia; CCS, Childhood Cancer Survivors; NSW, New South Wales; NT, Northern Territory; SA, South Australia; WA, Western Australia.

a Studies with a stated aim of investigating cancer in Indigenous Australian children or which made substantial findings related to cancer in Indigenous Australian children.

b Number of Indigenous children was derived from subtracting the number of non-Indigenous children from the total sample so this number may include children of unknown Aboriginal and Torres Strait Islander status.

**Table 2:** Descriptive studies.

Authors (Year of publication) location of study	Indigenous Australian childhood cancer focus^a^	Source of data	Date of study data	Study sample	Study aim	Relevant topics addressed in this review
Jessop & Phelan (2022)^23^ SA	Yes	Survey	Not stated	34 healthcare staff	Identify barriers in the understanding and provision of optimal palliative care to Indigenous Australian children with cancer by health-care staff.	Palliative care
Jessop et al. (2024)^24^ NT, SA, NSW	Yes	Interviews	2006–2021	9 families of children with cancer; 5 healthcare staff (2 ILO; 3 CNC)	Identify individual, systemic and cultural barriers to optimal healthcare for Indigenous Australian children with cancer.	Barriers
Shahid et al. (2011)^25^ WA	No	Interviews	2006–2007	30 Indigenous Australian adults affected by cancer; 1 participant spoke about their child with cancer	Investigate the experiences of Indigenous Australian people and barriers in accessing cancer services and treatment in Western Australia. To explore differences in experiences for Indigenous Australians based upon their residence in urban, rural or remote settings.	Barriers

CNC, Clinical Nurse Consultants; ILO, Indigenous Liaison Officer; NSW, New South Wales; NT, Northern Territory; SA, South Australia; WA, Western Australia.

a Studies with a stated aim of investigating cancer in Indigenous Australian children or which made substantial findings related to cancer in Indigenous Australian children.

Some studies were national in scope (n = 6), and others analysed data from South Australia (SA; n = 6), Western Australia (WA; n = 5), and the Northern Territory (NT; n = 5), with only one study including data from Victoria and one study including data from New South Wales (NSW). There were no studies from Queensland or Tasmania.

Most were observational epidemiological studies of national or state cancer registries, and hospital records (n = 13, 81%) (Table 1). Three papers were descriptive and reported findings from surveys or interviews with health care staff or families that made findings on Indigenous Australian children with cancer (Table 2). Almost half did not have a specific focus on Indigenous Australian children with cancer (n = 7, 44%); these were predominantly studies looking at childhood cancer across the whole population with a small number of findings and commentary related to findings on Indigenous Australian children with cancer. Only nine papers (56%) had a stated aim of investigating cancer in Indigenous Australian children or reported substantial findings related to cancer in Indigenous Australian children.

Eight papers reported on the incidence of childhood cancer in Indigenous Australian children, nine reported rates of mortality or survivor rates of childhood cancer in Indigenous Australian children, and four papers discussed barriers to diagnosis or treatment for Indigenous Australian children with cancer. No papers explored the psychosocial needs of Indigenous Australian children with cancer and no papers described interventions addressing psychosocial needs, although one study discussed palliative care for Indigenous Australian children with cancer from healthcare workers’ perspectives. The papers analysed various datasets with data collected between 1968 and 2021.

### Incidence

The most common types of cancers in Indigenous Australian children were leukemia and lymphoma,^17^ and melanoma was the least common type in Indigenous Australian adolescents.^12^ When considering all types of cancers, studies showed lower childhood cancer incidence rates in Indigenous Australian children compared to non-Indigenous children.^12^^,^^16^^,^^18^^,^^21^^,^^22^ However, one study found that the incidence ratio of childhood cancer of Indigenous Australian compared to non-Indigenous children had increased over time and noted that a plausible reason for the disproportionate increase was associated with improved identification of Indigenous Australian status.^22^

Studies exploring differences between age of diagnosis, sex and stages of diagnosis of cancers were limited. One study by Valery and colleagues analysed national Australian data between 1997 and 2007 and found no significant differences in the distribution of cancers by age, sex, and year of diagnosis between Indigenous Australian and non-Indigenous children.^19^ However, one study found that Indigenous Australian children with acute myeloid leukemia (AML) were younger at diagnosis than non-Indigenous children,^17^ while another found that Indigenous Australian children with acute lymphoblastic leukemia (ALL) were older at diagnosis than non-Indigenous children.^15^

The findings on incidence rates of types of cancer varied; some studies found no differences in the overall distribution of diagnostic groups of cancers,^19^^,^^22^ while other studies found different rates of specific types of cancer in Indigenous Australian children. Azzopardi and colleagues^12^ found lower rates of melanoma in Indigenous Australian adolescents compared to non-Indigenous adolescents. Studies have reported a proportional over-representation of AML in Indigenous Australian children in SA over the last 45 years. Bartle et al.^13^ noted an overrepresentation of AML when compared to ALL rates in Indigenous Australian children between 1979 and 1998 in SA (18% AML vs 1.2% ALL), Rotte et al.^17^ in their retrospective case note review of children with cancer in a tertiary hospital in SA from 1997 to 2011, which was the main referral centre for most children with suspected malignancies from the SA and NT, and found a significant over representation of AML in Indigenous Australian compared to non-Indigenous children (14.6% of Indigenous vs 4.1% of non-Indigenous children in the study sample). Similarly, Jessop et al.^15^ in their hospital records review for children with acute leukemia found a higher proportion of AML in Indigenous Australian children in SA and the NT between 2009 and 2018 (60.0% for Indigenous vs 14.4% for of non-Indigenous children in the study). However, differences were not found in WA between 2009 and 2018 in the same study.^15^

### Mortality

Brain cancers accounted for the most deaths followed by leukemias.^1^, ^18^ One study examined mortality rates of young Indigenous Australian children aged 1–6 years old and found that in contrast to non-Indigenous children, cancer was not one of the leading causes of death; rather, accidents, infections and birth defects had higher rates of mortality.^11^

Findings varied regarding whether Indigenous Australian children were more at risk of dying from cancer than non-Indigenous children. Some studies found no differences in mortality rates between Indigenous Australian and non-Indigenous children for cancers overall,^11^^,^^17^^,^^18^ while others found higher mortality rates in Indigenous Australian children than non-Indigenous children with cancer.^14^^,^^16^ However, it was noted by the latter studies that survival rates for Indigenous Australian children with cancer had improved over the last few decades.^14^^,^^16^

Valery and colleagues^19^ found a higher 5-year survival rate for non-Indigenous children than Indigenous Australian children, even after adjusting for rurality, socio-economic disadvantage, cancer diagnostic group, and year of diagnosis. This trend was also found regardless of stage of cancer (i.e., Stage I/II vs III+).^19^ A more recent study found similar poorer survival rates of Indigenous Australian children with cancer in earlier time periods, however noted that the differences in 5-year survival rates diminished over time.^22^

### Barriers identified for diagnosis and treatment

Remoteness of residence was a common barrier identified by studies for Indigenous Australian children in accessing cancer care.^15^^,^^17^^,^^24^ Other barriers identified included comorbidities, socioeconomic status, financial difficulties, psychosocial challenges, and systemic factors.^15^^,^^17^^,^^24^ Jessop and colleagues^24^ concisely summarised the barriers that Indigenous Australian children and families face in cancer diagnosis and treatment, classifying barriers in three categories: individual, systemic, and cultural barriers.

Studies have found that Indigenous Australian children were more likely to live in communities far from medical centres,^15^^,^^17^^,^^22^ have higher rates of hospitalisation,^10^ and longer stays in hospital than non-Indigenous children.^17^ Indigenous Australian children living in rural or remote areas often face difficulties accessing treatment in their communities, and challenges with relocating to metropolitan areas where tertiary hospitals are located.^24^^,^^25^ For example, relocating a child with cancer can have flow-on effects on the family, including a caregiver needing to relocate with (or without) siblings, impact on siblings’ schooling, childcare arrangements, accommodation and transport difficulties.^25^ Out-of-pocket expenses are particularly challenging for those living in rural areas who must travel to urban centres to receive cancer treatments which may extend over many weeks; families experience extra costs associated with accommodation, transport and food while living away from home.^23^^,^^24^ Challenges for single parents were also noted, particularly around balancing the needs of the ill child, care for siblings of the ill child, and the personal needs of the parent.^25^

Many Indigenous Australian families face challenges associated with having to travel long distances and being away from community and home for treatment; barriers identified included a lack of transport and accommodation for the family/siblings, delayed transfers, inflexibility of the health services’ Patient Assisted Transfer Scheme (PATS), identifying appropriate escorts for the child with cancer and resources for the escort, financial burden, a lack of physical and psychological preparation for families being away from community, and a lack of follow up services in remote communities.^24^ Other challenges include experiencing cultural insensitivity and poor cultural safety, poor explanation of medical-terms, and lack of involvement of key community and family members in appointments and decision making.^24^ Indigenous Australian children with cancer were more likely to be lost to follow-up than non-Indigenous children.^15^^,^^17^

Jessop and colleagues attributed delayed diagnosis of cancer in Indigenous Australian children to multiple intersecting challenges: geographic location, comorbidities, health literacy, financial constraints, kinship responsibilities of families, and systemic barriers for timely diagnosis (e.g., escalation pathways to tertiary hospitals, lack of paediatric specialists/social workers/Indigenous Liaison Officers).^24^ Comorbidities were identified as a barrier to receiving cancer care by two studies,^17^^,^^24^ but further details were not provided in the articles.

### Psychosocial needs and interventions

No studies exploring the psychosocial needs of Indigenous Australian children with cancer were identified, and no studies described interventions addressing psychosocial needs of Indigenous Australian children with cancer or their families.

Jessop and colleagues^23^ explored healthcare workers’ perspectives on palliative care for Indigenous Australian children with cancer, and found a lack of understanding and comfort among healthcare workers in caring for Indigenous Australian children with cancer at end-of-life. Health care workers reported barriers related to difficulties with communication, including variability in language/dialects, difficulties discussing end-of-life needs of the child and family, and differences in non-verbal communication.^23^ The study also identified that few staff had received education about the cultural needs of Indigenous Australian children at the end-of-life.^23^ Healthcare workers reported difficulties discharging Indigenous Australian children with cancer to rural and remote areas to die on Country,^23^ identifying issues with timing for discharge to Country and providing opportunities for cultural ceremonies if patients were unable to get home.

## Discussion

This study identified 16 peer-reviewed articles that reported on cancer in Indigenous Australian children. Most of the studies examined the incidence and mortality rates of childhood cancer, while no studies examined the psychosocial needs of Indigenous Australian children with cancer, and there were no psychosocial interventions for Indigenous Australian children with cancer. Some studies identified barriers to diagnosis and treatment. The relevance of the studies and gaps identified in the literature are discussed below.

### Incidence

When considering all types of cancers, studies showed lower childhood cancer incidence rates in Indigenous Australian children compared to non-Indigenous children.^12^^,^^16^^,^^18^^,^^21^^,^^22^ However, it is possible that the lower incidence rate was due to detection bias, as incidence ratios increased more in Indigenous Australian children compared to non-Indigenous children over the years, while identification of Indigenous Australian status improved.^22^ Studies conducted in SA found differences in incidence rates of different types of leukaemia in Indigenous Australian children collectively over four decades (AML vs ALL).^13^^,^^15^^,^^17^ However, differences in leukaemia presentations were not found in WA,^15^ and it was unclear whether differences were found for children in the NT, as data from SA and NT were combined in one study.^15^ It is possible that the differences in leukaemia presentations were associated with factors in SA that are different to other states (e.g., environmental, genetic); further research is needed to explore possible contributing factors.

Studies and reports in adult cancer in Australia showed higher prevalence of head, neck, lung and cervical cancers in Indigenous Australian compared to non-Indigenous adults,^1^^,^^26^^,^^27^ cancers that can be associated with modifiable risk factors, for example smoking and human papillomavirus (HPV) for which an effective vaccine now exists.^26^^,^^27^ However, childhood cancers differ from adult cancers in aetiology, biology, and treatment.^2^^,^^3^

Studies from other countries showed that risk of developing childhood cancer differs by race and ethnicity. For example, one study showed that most types of childhood cancer were higher in White than Black, Asian and Hispanic children, although it was unclear whether differences in incidence rates were associated with genetic or environmental factors.^28^ Another study showed significant racial and ethnic differences and age of presentations for various types of cancers including leukaemias, lymphomas, CNS tumours, blastomas, sarcomas, and germ cell tumours.^29^ Studies from around the world have suggested that genetic variants contribute to variations in presentations in cancers, for example, leukaemias,^30^, ^31^, ^32^ and neuroblastomas.^33^, ^34^, ^35^ More research is needed to explore whether differences in incidence rates of cancer are associated with genetic or environmental factors and to identify if there are modifiable risk factors for childhood cancer.

### Mortality

Findings varied regarding whether mortality rates in Indigenous Australian children with cancer were different to non-Indigenous children; some studies reported no significant differences in mortality rates,^11^^,^^17^^,^^18^ while others reported higher mortality rates in Indigenous Australian samples.^14^^,^^16^^,^^19^^,^^22^ In Australia, brain cancers were the leading cause of death in children with cancer.^1^ This was also the case for Indigenous Australian children, with brain cancers accounting for the most deaths followed by leukemias.^18^ There were no clear indications about the extent to which differences in mortality rates between Indigenous Australian and non-Indigenous children with cancer were a result of biological factors, differential access to diagnosis and treatment, or reflected other health behaviours.^14^

It was reported that survival rates for Indigenous Australian children with cancer had improved over the last few decades.^14^^,^^16^^,^^22^ However, there were no clear explanations in the literature about factors that contributed to the improved rates of survival, although this improvement is consistent with the efforts around Closing the Gap, which aimed at improving health outcomes for Indigenous Australians, including children.^36^ Better identification of Indigenous Australians, especially of children who might not have previously been identified as Indigenous Australian and potentially having a lifestyle and resources more akin to non-Indigenous people, could also appear as a decrease in the differential Indigenous/non-Indigenous survival rates.^37^

One paper argued that geographical remoteness, rather than Indigenous status, better explained the poorer outcomes of children with cancer, as poorer outcomes between those living in rural/regional areas and those in urban areas remained after controlling for Indigenous status.^20^ Geographic remoteness was associated with poorer childhood cancer survival rates, with children living in remote and very remote areas of Australia having worse cancer survival rates than those living in metropolitan areas.^14^^,^^15^^,^^19^^,^^20^ This differential in survival also occurs for adults with cancer in Australia and internationally.^38^, ^39^, ^40^, ^41^ As larger proportions of Indigenous Australian children live in rural areas compared to metropolitan areas, this also impacts data on Indigenous Australian child cancer outcomes.^15^^,^^17^^,^^19^ Causes of death in children with leukaemia living in remote areas was most often due to relapse or progression of the disease and infection.^15^ Poorer access to diagnostic and treatment services were suggested as likely causes of inferior survival rates for children living in remote areas, however, impact on survival of Indigenous Australian children with cancer is likely to be multifactorial.^14^^,^^19^ For example, two studies found that Indigenous Australian children were less likely to be enrolled in clinical trials,^15^^,^^17^ suggesting that Indigenous Australian children were less likely to receive advances in treatments that may be offered in clinical trials. In adults, differences in psychosocial and economic factors resulting in poorer access to healthcare are considered likely factors contributing to poorer outcomes of Indigenous Australian people with cancer.^42^^,^^43^ More research is needed in this area to understand the relationship between factors associated with risk of mortality from childhood cancers.

### Barriers identified for diagnosis and treatment

Many barriers for Indigenous Australian children with cancer were identified in the literature, including remoteness of residence, comorbidities, socioeconomic status, financial difficulties, psychosocial challenges, and systemic factors.^15^^,^^17^^,^^23^^,^^24^ These challenges have been described for adults with cancer and may be compounded when the patient is a child.^44^ As children are dependent on their caregivers to access medical care, access to treatment has a bi-directional relationship with the family's needs and resources; if the psychosocial and financial needs of the family are not addressed, the child with cancer faces significant challenges in receiving treatment following their diagnosis. Many Indigenous Australian families experienced cultural insensitivity and environments that felt culturally unsafe, poor explanation of medical-terms, and lack of involvement of key community and family members in appointments and decision making.^24^ Therefore, it is unsurprising that Indigenous Australian children with cancer were more likely to be lost to follow-up than non-Indigenous children.^15^^,^^17^ Inclusion of Indigenous Australian staff as part of cancer care can help to reduce miscommunications and patient stress, and can improve Indigenous Australian patient engagement.^44^^,^^45^

Comorbidities have been identified as a barrier to receiving cancer care.^17^^,^^24^ Indigenous Australian youth have higher levels of Type-2 diabetes than non-Indigenous youth,^46^, ^47^, ^48^ and infectious diseases are significantly more common in Indigenous Australian children than non-Indigenous children.^49^ Rotte and colleagues noted occurrences of hyperglycaemia during steroid therapy in Indigenous Australian children with cancer and speculated that this occurrence was associated with the high levels of insulin resistance in Indigenous Australian populations.^17^ Research on the relationships between comorbidities and treatment of cancer in Indigenous Australian children is currently lacking but is an important issue for consideration in future studies.

No publications were identified related to cancer treatment refusal and adherence in Indigenous Australian children. A systematic review of refusal of childhood cancer treatment identified four main reasons for treatment refusal by patients or patients’ decision makers: preference for alternative treatments, religious reasons, concerns about adverse effects of treatments, and a lack of insight into treatment needs.^50^ It is important to note that children rarely make decisions about their own healthcare, as they are intertwined in caregiver, family, and community dynamics. Many Indigenous Australian adults have reported wanting to use traditional medicines and healers, sometimes instead of or to supplement Western medical treatments,^51^ however the use of traditional Indigenous Australian medicines for childhood cancer is currently unknown.

No research papers that explored Indigenous Australians' perspectives on childhood cancer were identified, however research related to cancer in Indigenous Australian adults has identified the influence of cultural and psychosocial factors on cancer patients seeking medical attention.^42^^,^^51^ Some Indigenous Australian adults associated cancer with spiritual curses, believing that cancer was a punishment for a misdeed, and this resulted in fatalistic thinking and acceptance of diseases without seeking medical attention.^51^ Some Indigenous Australian adults reported their belief that cancer is a death-sentence, with disappointment in outcomes such as recurrence of cancer reinforcing mistrust in doctors or Western medicine.^51^ Indigenous Australian adults have also reported feeling isolated by friends and family who believed that cancer is contagious.^51^ Other psychosocial factors included feelings of shame, shyness with medical staff and not wanting to be a burden resulting in only seeking medical attention when feeling very sick.^42^ As children are often reliant on adult caregivers to receive medical attention, it is possible that these cultural and psychosocial factors are also relevant to Indigenous Australian children receiving a cancer diagnosis and treatment. Another barrier may be fear of involvement of government agencies such as child protection^52^; one study reported an example of a parent who was confronted with false allegations of child abuse after bringing their child to a hospital for medical care.^53^ Although not discussed in the identified studies, it is likely that parents and caregivers’ health literacy also impacts care-seeking, recognition of cancer symptoms, and decisions about treatment in Indigenous Australian children. More research is needed to identify and understand the unique barriers for Indigenous Australian children, so that paediatric services can provide appropriate supports and services to overcome these.

Remoteness of residence was a common barrier identified in the literature. Indigenous Australian children living in rural or remote areas often face difficulties accessing treatment in their communities, and challenges with relocation to metropolitan areas where tertiary hospitals are located.^24^^,^^25^ In the NT, children with complex cancer presentations need to relocate to a different state for paediatric care when treatment is not available locally.^54^ Jessop and colleagues have suggested a need for effective strategies to ensure that children in regional and rural areas receive appropriate service delivery and resources.^15^ These could include improvements in use of teleconferencing, using a shared care model, and deployment of outreach oncology nurses to improve communication with local health care workers. Given the importance of involvement of family and community in decision-making and the substantial barriers that Indigenous Australian children with cancer and their families face, it is important to optimise in-person paediatric cancer treatment and interventional services in regional and rural areas.

### Psychosocial needs and interventions

There was a lack of studies exploring psychosocial needs and interventions addressing psychosocial needs of Indigenous Australian children with cancer, although one study explored healthcare workers' perspectives on palliative care for Indigenous Australian children. The study identified systemic barriers to meeting the needs of Indigenous Australian children with cancer by healthcare workers in palliative care, including a lack of education about the cultural needs of Indigenous Australian children at the end-of-life, and challenges with discharging Indigenous Australian children with cancer to rural and remote areas to die on Country.^23^ For many Indigenous Australians, the place of death is culturally and spiritually significant, and for many families it is important to have their loved one die at home or on Country.^55^ While this was understood by the healthcare workers in the study, some reported fear of raising ‘forbidden’ subjects and commented on the challenges in communicating prognosis and futility concepts.^23^

Culturally secure palliative care services are an important part of cancer care for Indigenous Australian children. Although Jessop and Phelan's^23^ study analysed healthcare workers' perspectives on palliative care, the questionnaire was co-designed by Indigenous Australian people and identified concepts important in Indigenous Australian cultures. In addition to illustrating specific psychosocial needs that should be investigated through future studies with Indigenous Australians, the concepts explored in Jessop and Phelan's^23^ paper are important in informing new models of palliative care services for Indigenous Australian children with cancer. Important factors to consider included language and communication challenges, end-of-life customs, mourning and grieving rituals, talking about the concept of death and spirituality, use of traditional medicines, and the involvement of family and community at end-of-life,^23^ concepts consistent with those identified in research with Indigenous Australian adults.^25^^,^^51^^,^^55^^,^^56^ In order to provide adequate palliative care for Indigenous Australian children, services must have the resources to deliver complex medical care and ensure culturally safe practices, particularly for conversations around death and dying.^57^ Future studies may also consider using advance care planning tools to ensure that interventions and palliative care services are patient-centred; for example, Evans and colleagues^58^ adapted the Voicing My CHOiCES tool for Australian adolescents and young adults with life-limiting illnesses. Future studies may consider adapting such tools to be culturally and literacy-level appropriate for Indigenous Australian children.

Schilstra and colleagues^59^ conducted an internet search and found no culturally inclusive resources that support Indigenous Australian youth with cancer to return to study or work. Despite the absence of literature about the psychosocial needs or interventions for Indigenous Australian children with cancer, studies exploring the psychosocial needs of non-Indigenous children with cancer and Indigenous Australian adults with cancer provide important concepts for consideration. Given the small numbers of Indigenous Australian children with cancer, it is likely that collaborative research across Australia could improve the collection of quality information from Indigenous Australian children with cancer and their caregivers to elucidate differences and commonalities in beliefs, needs and experiences across the diversity of Indigenous Australians.

### Learnings from non-Indigenous studies about children with cancer

Studies exploring the psychosocial needs of children with cancer identified needs relating to hospital spaces, psychological and emotional support, social and peer support, family needs, educational needs, and managing side effects of treatment.^60^^,^^61^ Hospital environments need to foster comfort and inclusivity through having open spaces and access to nature, provision of food tailored to the interests and needs of the child, a variety of resources for entertainment, comfortable bedrooms, and spaces available for caregivers and families to rest^60^; the same needs as were identified in relation to planning of services for Indigenous Australian people with cancer.^44^ Maintaining social connection was also identified as an important need: being able to connect with peers, friends and family members, receiving social support from communities and schools, and educating peers about cancer to prevent insensitive questions directed at the child with cancer.^60^^,^^61^ Other psychosocial needs included support in social skills training, education about sexual health, and spiritual needs.^60^ School-related needs included support for keeping up with schoolwork, transitioning back to school, and concentration.^61^

Research has also shown the importance of providing support to caregivers of children with cancer. Caregivers' emotional struggles have been shown to impact their child's cancer treatment, ability to provide support to the child and to care for the siblings of the ill child; challenges which over time may contribute to family conflict.^62^^,^^63^ Therefore, interventions should be facilitated to optimise parent, child and family well-being.^64^ Cognitive behaviour therapies have been found to be effective at improving quality of life and reducing distress for children with cancer and their families.^65^ Support needs of caregivers included coaching for parents in managing externalising behaviours, navigating difficult conversations, education about the cancer diagnosis and nutrition, managing dynamics with siblings of the child with cancer, and financial support.^60^

Kazak and colleagues^66^ emphasised the importance of psychosocial assessment of children and families, and recommended it be integrated as a standard of care for youth diagnosed with cancer. Even though many families cope and adjust to their child's cancer diagnosis and treatment, it is important to identify those individuals and families that continue to experience difficulties so that adequate supports can be provided to prevent long-term adverse outcomes.^66^ The extent to which these needs are met and managed for Indigenous Australian children with cancer, especially for children living in regional or remote areas or with complex family situations, is currently unknown as no published literature was identified.

### Learnings from studies with Indigenous Australian adults with cancer

There are considerable learnings from research regarding Indigenous Australian adults with cancer. Valery and colleagues classified psychosocial needs into four categories: hospital care, physical/psychological, practical/cultural, and information/communication needs.^67^ Indigenous Australian adults with cancer indicated needs which included support managing physical pain, impact of cancer on their daily functioning, emotions such as anxiety and stress about cancer prognosis and mortality, financial concerns, and finding accommodation when relocating for treatment.^56^

Culture and family are particularly important factors to consider when treating Indigenous Australian adults with cancer, with family often providing practical and emotional support.^68^ Therefore, it is important to provide families with an available space to gather, include relevant people in decision making processes, provide updates and logistical support such as organising transport or accommodation for family.^69^ Having an Indigenous Australian person to talk to and support the person with cancer was a need identified for adults with cancer.^56^ Indigenous health professionals and Indigenous cancer navigators were found to improve the cultural safety of cancer care, improve communication, advocate for patients and educate non-Indigenous staff.^45^

Future interventions for Indigenous Australian children should consider the above concepts from studies exploring the psychosocial needs of non-Indigenous children with cancer and Indigenous Australian adults with cancer. These concepts include addressing hospital-related, environmental, physical, psychological, social, practical, cultural, educational, and communication needs.

## Limitations

We are aware that not all advances are captured in peer reviewed literature, and that there are unpublished research studies and ongoing projects relating to childhood cancer in Indigenous Australians. Current and ongoing improvements in practice may not be captured in the literature. This paper has focussed on published peer-reviewed articles and even that literature is limited, with many studies having small samples of Indigenous Australian children. Some papers included in this review included children, adolescents and young adults,^12^^,^^14^^,^^16^ yet the conclusions have been generalised to children/youth. Future studies may consider the different needs of children, adolescents and young adults, and the differences in services available between paediatric and adult services.

## Limitations of the literature

There are considerable challenges in conducting research with Indigenous Australian children with cancer due to practical barriers and small numbers; Indigenous Australians represent only 3.2% of Australia's population,^70^ and childhood cancer is a rare disease. Due to small sample sizes (samples of Indigenous children with cancer in identified studies ranged from 0.06%–36%), it is difficult to make generalisations about Indigenous Australian children with cancer, particularly with respect to different types of cancers. It is also noted that identification of Indigenous Australian status has historically been poor and while this has improved significantly in recent years, poor identification may have influenced the findings in studies of cohorts in earlier decades.

Most studies identified for this article used data from WA, SA, and the NT. This may reflect historical challenges and differences with Indigenous status identification in different jurisdictions of Australia and the need to aggregate data across many years in order to assess differentials in rates of relatively rare diseases and outcomes.^37^^,^^71^ However, since Indigenous Australian peoples are diverse, the needs of children with cancer and their families may differ across Australia and have changed over time. It is important to note that Indigenous Australians experience challenges besides those relating to health, for examples interactions with the justice system, racism, social-economic disadvantage, and reduced access to services, education, and technology; these issues are likely to have compounding effects on Indigenous Australians’ health and cancer outcomes. Conducting research and implementing services to manage complex conditions in rural and remote areas is challenging due to costs, workforce availability, required skills of specialised workers, and resources required such as machinery for scans and treatments.

Conducting qualitative research with children is challenging, as children are not able to provide informed consent to participate without parental consent, young children are not able to articulate their experiences, and interviews with parents will be inevitably biased towards adult perspectives. Although children are dependent on their caregivers, children and adolescents are not small adults and have different needs from adults.^72^ Despite the challenges, future studies should consider creative ways to gain children's perspectives to develop services that are appropriate for their needs.

## Gaps identified for future research to address

Current published papers have mainly explored incidence and mortality rates in Indigenous Australian children with cancer. Although understanding epidemiological trends are important, there is a lack of studies exploring many important issues related to Indigenous Australian children with cancer. These include underlying factors relating to the presentation and treatment of cancer in Indigenous Australian children (e.g., biological, environmental and behavioural factors), survival outcomes (e.g., progression/remission of cancer, complications such as infections), the attitudes and beliefs of Indigenous Australian people about childhood cancer and treatment, experiences of Indigenous Australian children with cancer, care-givers’ experiences of caring for Indigenous Australian children with cancer, psychosocial needs and priorities of Indigenous Australian children with cancer, culturally safe interventions that address the psychosocial needs of Indigenous Australian children with cancer, challenges experienced when transitioning from paediatric care to adult services, and the long-term effects of cancer diagnosis and treatment on Indigenous Australian children (e.g., impacts on mental health, physical health, effects of pharmacological treatments, and fertility as adults). These issues are all worthy of further study and would be strengthened by larger numbers through national collaboration. Children with cancer have been shown to have higher psychiatric hospitalisations, community mental health service contacts, and a higher risk of mental health difficulties compared to children without cancer,^73^ so future studies with Indigenous Australian children with cancer have many factors to consider, including addressing hospital-related, environmental, physical, psychological, social, practical, cultural, educational, and communication needs.

## Conclusions

The lack of studies about Indigenous Australian children with cancer was noted more than ten years ago,^17^ and progress in filling this gap has been slow. While the literature about Indigenous Australian children with cancer is still lacking, this review has identified studies exploring incidence and mortality rates, and identified barriers to diagnosis and treatment. The literature showed lower overall cancer incidence rates in Indigenous Australian children compared to non-Indigenous children, however, this finding may be associated with detection bias due to under ascertainment of Indigenous Australian status. It was unclear whether there were differences in mortality rates in Indigenous Australian compared to non-Indigenous children with cancer, although studies reported an improvement in survival rates of Indigenous Australian children with cancer over the last few decades. Barriers to diagnosis and treatment consisted of individual, systemic and cultural factors, including remoteness of residence, comorbidities, socioeconomic status, financial difficulties, psychosocial challenges and difficulties related to healthcare service delivery. This review highlights the lack of published knowledge about psychosocial needs and interventions for Indigenous Australian children with cancer and their families. While some findings relating to Indigenous adults may be relevant for Indigenous children, there may be important differences as children have different needs to adults.

This review has identified that considerable additional research is needed in this field. While it is encouraging that research in this field is increasing, future research should seek to understand the intersecting biological, environmental, behavioural, and psychological factors that influence outcomes of cancer in Indigenous Australian children. Research is urgently needed to identify and support the psychosocial needs of Indigenous Australian children and their families, and develop relevant holistic interventions that can improve physical and psychological wellbeing in the long-term. Given the challenges identified in this paper of conducting research with Indigenous Australian children, a collaborative approach across the country may be a helpful strategy to overcome the limitations of small samples. Achieving better cancer outcomes for Indigenous Australian children requires co-designed and collaborative research projects, with an emphasis on rigorous evaluation and the sharing of findings to strengthen future interventions.

## Contributors

The research team consisted of five people (SB, EVT, MR, LP and SCT). MR has cultural connections to the Gidja and Ngarinyin people of the Kimberley in Western Australia, and extensive clinical and research experience in improving outcomes for Indigenous Australian children. LP is a Nyoongar, Palyku woman with ties to the Martu people in the Western Desert, a passionate advocate for improving cancer outcomes in Indigenous Australians, and has held multiple clinical, advisory and research roles. The three non-Indigenous team members have a combined experience with collaborative research into improving Indigenous Australian health outcomes of over thirty years, including many years of research around Indigenous Australians and cancer, primarily with the focus on adults and often with a focus on health services.

Conceptualization: SB, EVT, MR, LP, SCT.

Data curation: SB, EVT.

Formal analysis: SB, EVT.

Investigation: SB, EVT.

Methodology: SB, EVT.

Project administration: SB, SCT.

Visualization: EVT, SB.

Writing—original draft: SB.

Writing—review & editing: SB, EVT, MR, LP, SCT.

## Data sharing statement

Not applicable, there is no primary data for this paper.

## Declaration of interests

SCT is a member of the Aboriginal Advisory Committee of the Cancer Council of Western Australia, however this membership was not associated with this manuscript. All other authors declare no conflicts of interest.
