# Supplementary material for: Aboriginal and Torres Strait Islander children and cancer: a narrative review of incidence, mortality, barriers to diagnosis and treatment, psychosocial needs and interventions
Source: Lancet Reg Health West Pac. 2025 Mar 30;61:101530. doi: 10.1016/j.lanwpc.2025.101530 (PMC12414359; doi:10.1016/j.lanwpc.2025.101530)
Supplement: Appendix A [file mmc1.docx]

**Appendix A:**

**Search terms used in databases**

**PubMed** (Search conducted: 9 May 2024)

| Concept terms | | |
| --- | --- | --- |
| 1 | Australian Aboriginal and Torres Strait Islander Peoples[mh] OR Indigenous[tiab] OR Aboriginal[tiab] OR "Torres Strait Islander"[tiab] OR "Torres Strait Islanders"[tiab] OR "First Nations"[tiab] OR "First Australians"[tiab] | 54,168 |
| 2 | Child[mh] OR Pediatrics[mh] OR Infant[mh] OR paediatric[tiab] OR pediatric[tiab] OR child*[tiab] OR youth[tiab] OR adolescent[tiab] | 3,528,618 |
| 3 | Neoplasms[mh] OR cancer[tiab] OR oncology[tiab] | 4,647,922 |
| 4 | Australia[mh] OR Australia*[tw] OR "northern territory" [tiab] OR "northern territory" [ad] OR tasmania[tw] OR tasmania[ad] OR "new south wales" [tw] OR "new south wales" [ad] OR victoria[tw] OR victoria[ad] OR queensland[tw] OR queensland[ad] | 597,429 |
|  | **1 AND 2 AND 3 AND 4** | **63** |

**CINAHL** (Search conducted: 9 May 2024)

| Concept terms | | |
| --- | --- | --- |
| 1 | Indigenous OR Aboriginal OR "Torres Strait Islander" OR "Torres Strait Islanders" OR "First Nations" OR "First Australians" | 49,136 |
| 2 | Paediatric* OR pediatric* OR child* OR youth OR adolescen* | 1,362,747 |
| 3 | Neoplasm* OR cancer* OR oncology | 786,772 |
| 4 | Australia* OR "Northern Territory" OR Tasmania OR "New South Wales" OR Victoria OR Queensland | 163,057 |
|  | **1 AND 2 AND 3 AND 4** | **76** |

**PsycInfo** (Search conducted: 9 May 2024)

| Concept terms | | |
| --- | --- | --- |
| 1 | Indigenous OR Aboriginal OR "Torres Strait Islander" OR "Torres Strait Islanders" OR "First Nations" OR "First Australians" | 21,179 |
| 2 | Paediatric* OR pediatric* OR child* OR youth OR adolescen* | 1,260,080 |
| 3 | Neoplasm* OR cancer* OR oncology | 92,125 |
| 4 | Australia* OR "northern territory" OR tasmania OR "new south wales" OR victoria OR queensland | 72,260 |
|  | **1 AND 2 AND 3 AND 4** | **16** |

**Australian Indigenous HealthInfoNet** (Search conducted: 9 May 2024)

This database includes publications and grey literature about Australian Aboriginal and Torres Strait Islander health and wellbeing, therefore concept 1 terms “Indigenous OR Aboriginal” and 4 “Australia” were not necessary in the search of this database.

| Concept terms | | |
| --- | --- | --- |
| 2 | Children or adolescents | |
| 3 | Cancer | |
|  | **2 AND 3** | **15** |

**Google Scholar** (Search conducted: 14 May 2024)

Authors performed a title and abstract review until page 10 (first 100 results). No relevant articles were retrieved after the first 80 results. The search retrieved 14 additional articles that had not previously been identified in other database searches.

| Concept terms | | |
| --- | --- | --- |
| 1 | Indigenous OR Aboriginal | |
| 2 | Paediatric OR child* OR youth OR adolescent | |
| 3 | Cancer | |
| 4 | Australia | |
|  | **1 AND 2 AND 3 AND 4** | **90,600** |
|  | **Included for full text review** | **14** |
